# Effect of duloxetine in patients with fibromyalgia: tiredness subgroups

**DOI:** 10.1186/ar3081

**Published:** 2010-07-14

**Authors:** Laurence A Bradley, Robert Bennett, Irwin J Russell, Madelaine M Wohlreich, Amy S Chappell, Fujun Wang, Deborah N D'Souza, Harvey Moldofsky

**Affiliations:** 1 Division of Clinical Immunology and Rheumatology, University of Alabama at Birmingham, 177A Shelby Interdisciplinary Research Building, 1825 University Boulevard, Birmingham, Alabama 35294, USA; 2 Oregon Health & Science University, 3455 SW US Veterans Hospital Road, Portland, OR 97239, USA; 3 The University of Texas Health Science Center at San Antonio, 7703 Floyd Curl Drive, San Antonio, TX 78229, USA; 4 Lilly Research Laboratories, Lilly Corporate Center, Indianapolis, IN 46285, USA; 5 Medical, Neuroscience, Lilly Research Laboratories, Lilly Corporate Center, Indianapolis, IN 46285, USA; 6 IU The Indiana University Medical Center, Department of Neurology, 545 Barnhill Drive, Indianapolis, IN 46202, USA; 7 Statistics, Neuroscience, Lilly Research Laboratories, Lilly Corporate Center, Indianapolis, IN 46285, USA; 8 Scientific Communications, Lilly Research Laboratories, Lilly Corporate Center, Indianapolis, IN 46285, USA; 9 The Toronto Psychiatric Research Foundation, 951 Wilson Avenue, Toronto, Ontario, M3K 2S7, Canada

## Abstract

**Introduction:**

This study tested the hypothesis that baseline ratings of fatigue/tiredness would be negatively associated with the efficacy of duloxetine on measures of pain and functional ability in patients with fibromyalgia.

**Methods:**

A post hoc analysis of pooled data from 4 double-blind, placebo-controlled studies of duloxetine in fibromyalgia was performed. The fibromyalgia impact questionnaire (FIQ) tiredness item score (0 to 10 scale) was used to define tiredness subgroups. Patients were stratified into 3 subgroups: mild (0 to 3), moderate (4 to 6), and severe (7 to 10) tiredness. Analysis of covariance models and logistic regressions were used to test treatment-by-tiredness subgroup interactions.

**Results:**

Data from the first 3 months are included in this post hoc analysis (duloxetine N = 797, placebo N = 535). At baseline, the distribution of tiredness severity in the duloxetine and placebo groups respectively was 3.64% and 3.75% mild, 16.71% and 15.57% moderate, and 79.65% and 80.68% severe. Rates of clinically significant (≥30% and ≥50%) improvement in brief pain inventory (BPI) average pain were similar across the tiredness subgroups. Tiredness severity at baseline was not negatively associated with the effects of duloxetine on patients' reports of functional ability using the FIQ total score, FIQ measures of physical impairment, interference with work, pain, stiffness, and depression and the medical outcomes study short form-36 (SF-36).

**Conclusions:**

Studies of duloxetine in fibromyalgia have demonstrated clinically significant improvements in pain and functional ability (FIQ, SF-36). This post hoc analysis of data shows that the efficacy of duloxetine among patients with fibromyalgia does not vary as a function of baseline ratings of fatigue/tiredness.

## Introduction

Fibromyalgia is a disorder that is found in approximately 2% of the general population and is characterized by chronic widespread pain and tenderness [1]. Additional symptoms that often accompany fibromyalgia are tiredness or fatigue, depressed mood, sleep disturbance, and headache [2,3]. We will use the term 'tiredness' throughout this report, due to our decision to use the fibromyalgia impact questionnaire (FIQ) outcome measure, unless we refer specifically to studies that have used measures of 'fatigue'.

Duloxetine, a potent reuptake inhibitor of serotonin and norepinephrine, was approved in the USA for the management of fibromyalgia in June 2008. Preclinical models of central sensitization suggest that duloxetine is efficacious in the treatment of patients with persistent/chronic pain. In rodents, duloxetine has demonstrated efficacy in the formalin and capsaicin models of persistent pain, as well as in the partial sciatic nerve ligation and L5/L6 spinal nerve ligation models of neuropathic pain [4,5]. Duloxetine is efficacious in the treatment of the painful physical symptoms associated with depression and the pain associated with diabetic neuropathy in nondepressed patients [6,7]. The results of four placebo-controlled clinical trials of duloxetine in the treatment of fibromyalgia have been previously reported [8-11].

Patients with fibromyalgia frequently report moderate to severe fatigue and the prevalence of fatigue in patients with fibromyalgia (76%) is almost double that of patients with osteoarthritis or rheumatoid arthritis (41%) [12,13]. An evidence-based review of the literature on chronic pain found a highly consistent association between fatigue and pain in multiple studies and concluded that this may represent an etiological relation [14]. This conclusion was based in part on the results of several studies showing that fatigue develops following the onset of chronic pain and that severity of chronic pain is positively associated with the likelihood of subsequent reports of fatigue [15,16]. However, few studies have assessed whether fatigue or tiredness levels are associated with variations in the efficacy of pharmacologic therapies for chronic pain. This is a particularly important issue in the management of patients with fibromyalgia who often report relatively high levels of fatigue/tiredness in addition to chronic widespread pain. The present study analyzed data from four placebo-controlled trials of duloxetine in patients with fibromyalgia to test the hypothesis that baseline ratings of tiredness would be negatively associated with the efficacy of duloxetine on measures of pain and functional ability in patients with fibromyalgia.

## Materials and methods

This study comprised a pooled analysis of four double-blind, placebo-controlled, randomized, multicenter clinical trials of duloxetine in patients with fibromyalgia. These studies were conducted in accordance with the ethical principles that have their origin in the Declaration of Helsinki and that are consistent with good clinical practices and the applicable laws and regulations. Table 1 shows the dosage, administration, and duration of treatment for each of the studies. The details of these studies have been reported previously [8-11] and are summarized here. Male and female patients were entered into studies 1, 3, and 4, whereas study 2 included only female patients. Only three-month data are included in the analyses presented here.

**Table 1:** Summary of placebo-controlled trials of duloxetine for the treatment of fibromyalgia

Study	**Treatment duration**^**a **^**(weeks)**	Placebo (n)	Dosage	Duloxetine (n)
Arnold and colleagues 2004 [8]	12	103	60 mg BID	104
Arnold and colleagues 2005 [9]	12	120	60 mg QD	118
			60 mg BID	116
			20 mg QD	79
Russell and colleagues 2008 [11]	28	144	60 mg QD	150
			120 mg QD	147
Chappell and colleagues 2008 [10]	28	168	60/120 mg QD	162

^a^Three-month data are included in the analyses presented. BID, twice daily; QD, once daily.

Included patients were at least 18 years of age and met the criteria for fibromyalgia as defined by the American College of Rheumatology. In study 1, patients were required to indicate a pain score of at least 4 on the FIQ pain item (score range of 0 to 10, with 10 indicating very severe pain) [17]. In studies 2 to 4, patients were required to indicate a pain score of at least 4 on the 24-hour average pain severity item of the brief pain inventory (BPI) (score range of 0 to 10, with 10 indicating pain as bad as you can imagine) [18]. The major exclusion criteria were serious or unstable medical or psychiatric illness, current primary psychiatric diagnosis other than major depressive disorder, a primary diagnosis of anxiety disorder in the past year, pain from traumatic injury, or rheumatologic illness [8-11].

All four studies assessed the effects of duloxetine and placebo on the BPI average pain score, the FIQ (a patient self-reported instrument that assesses the impact of fibromyalgia symptoms and functional impairment; the FIQ total score ranges from 0 (no impact) to 80 (maximum impact)), the patient global impressions of improvement (PGI-Improvement, a patient-rated global assessment of response to treatment with scores ranging from 1 (very much better) to 7 (very much worse)), and the medical outcomes study short form-36 (SF-36, a quality-of-life measure including eight health-status domains that are each scored 0 to 100, with higher scores indicating better health).

The raw scores for the FIQ tiredness item range from 0 to 10 [17]. In the analyses reported here, the FIQ tiredness item is used to substitute for a formal measure of fatigue (multidimensional fatigue inventory), because the latter was not used in all the four fibromyalgia studies. Based on the FIQ tiredness item score (0 to 10) at study entry, patients were stratified into three subgroups: mild (0 to 3), moderate (4 to 6), or severe (7 to 10) tiredness. Previous studies have also used an 11-point numeric rating scale for measuring fatigue, such as the fatigue severity scale [19] and the brief fatigue inventory where scores within the ranges of 0 to 3, 4 to 6, and 7 to 10 points indicate mild, moderate, and severe fatigue levels, respectively [20].

Treatment-emergent adverse events (TEAEs), collected at every visit by spontaneous reporting from the patient, within the three subgroups were also evaluated. A TEAE was any reported event that first occurred or worsened in severity during treatment, compared with the predefined baseline period.

### Statistical methods

All randomized patients (except study 3 patients on the suboptimal dose of 20 mg once daily (*n *= 79) with at least one nonmissing postbaseline assessment were included in the efficacy analyses.

Changes from baseline to endpoint for continuous efficacy measures were analyzed using an analysis of covariance model, with missing values imputed using the last-observation-carried-forward approach. The model included baseline, treatment, study, tiredness subgroup, and treatment-by-tiredness subgroup interaction.

The baseline characteristics were analyzed using the Cochran-Mantel-Haenszel test controlling for study, or analysis of variance, which included treatment and study.

Categorical outcomes were analyzed using logistic regression (including terms for study, treatment, tiredness subgroup, and treatment-by-tiredness subgroup interaction) or Fisher's exact test. Treatment effects were tested at a two-sided significance level of 0.05. Interaction effects were tested at a two-sided significance level of 0.1. The term 'significant' indicates statistical significance throughout the manuscript.

## Results

### Demographics

Across all studies, 797 patients received duloxetine and 535 patients received placebo for approximately three months. There were no significant differences between duloxetine-treated and placebo-treated patients in any of the patients' demographic variables. The mean age of patients was 50.2 years (standard deviation = 11.0, range 19 to 83 years); the majority of patients were female (n = 1262, 94.7%) and Caucasian (n = 1169, 87.8%).

### Distribution of tiredness subgroups

At baseline, the distribution of tiredness severity in the duloxetine group (based on the FIQ tiredness item score) was 3.64% (n = 29) mild (0 to 3), 16.71% (n = 133) moderate (4 to 6), and 79.65% (n = 634) severe (7 to 10); the distribution in the placebo group was 3.75% (n = 20) mild, 15.57% (n = 83) moderate, and 80.68% (n = 430) severe. Within the duloxetine and placebo groups, the mean baseline FIQ tiredness scores in the mild subgroup were 2.5 and 2.7, respectively, in the moderate subgroup was 5.3 (duloxetine and placebo), and in the severe subgroup were 8.6 and 8.7, respectively.

### Efficacy measures by tiredness subgroups

#### Brief pain inventory

**Figure 1:**
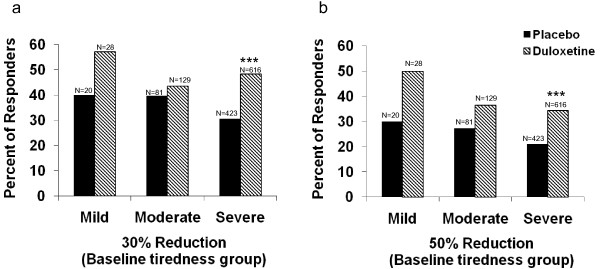
**Brief pain inventory response**. Proportions of patients with **(a) **30% and **(b) **50% reduction in the average brief pain inventory score. ***P < 0.001 vs. placebo. With both of the outcome measures, between-groups (duloxetine-treated vs. placebo-treated) analysis showed that the group of patients receiving duloxetine exhibited significantly greater numbers of responding patients than among those receiving placebo, but this was only true among the patients in the more severe tiredness group. There was no significant treatment-by-tiredness subgroup interaction between the three subgroups (*P *> 0.1).

Within the severe subgroup, there was a significant difference between the duloxetine and placebo groups (*P *< 0.001) in the number of patients with 30% or greater and 50% or greater reductions in the BPI average pain response (Figure 1). For the BPI average pain score, within the severe subgroup there was a significant difference (*P *< 0.001) in the mean change from baseline to endpoint between the duloxetine and placebo groups (Table 2).

**Table 2:** Efficacy and functional outcome measures

	Mild subgroup				Moderate subgroup				Severe subgroup			
	Placebo		Duloxetine		Placebo		Duloxetine		Placebo		Duloxetine	
	BL Mean (SD)	Change Mean (SE)	BL Mean (SD)	Change Mean (SE)	BL Mean (SD)	Change Mean (SE)	BL Mean (SD)	Change Mean (SE)	BL Mean (SD)	Change Mean (SE)	BL Mean (SD)	Change Mean (SE)
**BPI average Pain score**	4.5 (1.3)	-1.8 (0.6)	5.6 (1.7)	-1.3 (0.5)	5.5 (1.3)	-1.1 (0.2)	5.5 (1.4)	-1.6 (0.2)	6.7 (1.5)	-1.1 (0.1)	6.6 (1.5)	-2.0 (0.1)***
												
**FIQ**												
Total score	27.3 (11.4)	-1.0 (4.2)	26.4 (7.6)	1.0 (3.5)	40.1 (8.8)	-4.4 (1.7)	39.3 (9.6)	-7.7 (1.3)	54.9 (9.9)	-9.4 (0.8)	54.4 (10.8)	-14.4 (0.7)***
Physical Impairment score	1.8 (1.8)	-0.3 (0.6)	2.5 (1.9)	0.1 (0.5)	3.3 (2.0)	-0.2 (0.2)	3.2 (2.2)	-0.6 (0.2)	4.7 (2.1)	-0.4 (0.1)	4.8 (2.3)	-0.8 (0.1)*
Interference with work	3.0 (2.2)	-0.6 (0.7)	3.4 (2.2)	-0.0 (0.5)	5.0 (1.7)	-0.9 (0.3)	4.8 (2.3)	-1.3 (0.2)	6.9 (1.9)	-1.2 (0.1)	6.8 (2.2)	-2.0 (0.1)***
Pain	4.1 (2.4)	-0.7 (0.7)	5.2 (2.4)	-1.0 (0.6)	6.0 (1.6)	-0.9 (0.3)	6.2 (1.7)	-1.5 (0.2)	7.6 (1.7)	-1.4 (0.1)	7.5 (1.7)	-2.1 (0.1)***
Tiredness	2.7 (0.6)	1.6 (0.8)	2.5 (0.7)	2.4 (0.7)	5.3 (0.8)	0.1 (0.3)	5.3 (0.8)	-0.2 (0.2)	8.7 (1.1)	-1.6 (0.1)	8.6 (1.1)	-1.8 (0.1)
Rested	3.5 (2.0)	0.8 (0.7)	3.9 (1.7)	0.7 (0.6)	6.4 (1.9)	-0.9 (0.3)	6.3 (2.1)	-1.0 (0.3)	8.4 (1.6)	-1.5 (0.1)	8.4 (1.7)	-1.8 (0.1)
Stiffness	4.4 (2.4)	0.7 (0.7)	5.2 (2.6)	-1.5 (0.5)	6.5 (2.0)	-1.4 (0.3)	6.6 (2.0)	-1.8 (0.2)	8.1 (1.7)	-1.6 (0.1)	8.0 (1.7)	-2.5 (0.1)***
Anxious^a^	2.7 (2.8)	-0.1 (0.6)	1.4 (1.9)	0.3 (0.5)	3.7 (2.3)	-0.6 (0.3)	3.1 (2.6)	-0.8 (0.2)	5.2 (3.0)	-0.8 (0.1)	5.0 (3.2)	-1.8 (0.1)***
Depression	1.2 (1.4)	0.2 (0.5)	1.3 (1.8)	0.2 (0.4)	2.5 (2.4)	-0.2 (0.2)	2.4 (2.5)	-0.8 (0.2)*	4.5 (3.0)	-0.5 (0.1)	4.4 (3.2)	-1.6 (0.1)***
												
**SF-36**												
Mental component summary	55.2 (7.7)	-0.9 (2.4)	52.9 (8.0)	0.3 (2.0)	49.2 (10.3)	-0.1 (1.0)	51.1 (10.5)	1.6 (0.8)	42.7 (11.1)	2.0 (0.5)	42.8 (11.8)	5.4 (0.5)***
Physical component summary	33.8 (6.2)	3.8 (2.0)	33.9 (9.3)	2.8 (1.7)	31.6 (7.6)	2.5 (0.9)	31.2 (8.3)	4.2 (0.8)	27.6 (7.4)	3.1 (0.4)	27.8 (7.6)	4.1 (0.4)
Bodily pain	42.7 (8.4)	6.9 (4.8)	41.1 (17.8)	15.7 (3.9)	37.7 (13.2)	6.2 (2.1)	36.7 (12.2)	12.3 (1.7)*	27.8 (13.6)	8.6 (0.9)	28.4 (13.6)	14.2 (0.8)***
General health	60.1 (14.7)	3.5 (2.6)	58.9 (20.1)	6.9 (2.1)	53.3 (21.1)	0.7 (1.7)	53.9 (19.8)	4.1 (1.4)	41.8 (20.0)	5.1 (0.8)	44.0 (21.0)	7.7 (0.7)*
Mental health	75.3 (16.5)	1.3 (3.5)	77.2 (13.5)	3.5 (2.8)	69.5 (17.4)	1.8 (1.7)	72.6 (17.0)	4.6 (1.4)	60.9 (19.2)	3.3 (0.9)	61.2 (20.8)	9.9 (0.7)***
Physical functioning	53.1 (19.1)	9.4 (4.4)	57.2 (21.6)	8.0 (3.5)	51.3 (21.2)	6.9 (2.1)	48.9 (20.4)	8.0 (1.7)	40.2 (21.1)	5.7 (0.9)	39.4 (21.8)	9.5 (0.8)**
Role-emotional	88.9 (28.0)	-2.4 (8.4)	75.6 (37.2)	-1.5 (6.9)	69.3 (39.0)	1.1 (4.0)	71.8 (37.0)	4.7 (3.2)	50.0 (42.6)	5.5 (2.0)	48.4 (43.8)	16.1 (1.7)***
Role-physical	44.4 (34.9)	6.1 (8.8)	37.5 (41.4)	-6.8 (7.2)	25.0 (34.1)	5.7 (4.2)	30.6 (36.9)	12.7 (3.4)	13.0 (23.1)	8.7 (1.7)	12.5 (23.6)	13.6 (1.4)*
Social functioning	79.2 (23.1)	3.8 (4.7)	77.3 (19.6)	0.0 (3.8)	68.0 (22.5)	4.0 (2.2)	69.6 (25.0)	7.4 (1.7)	51.0 (23.3)	7.6 (1.1)	52.3 (24.8)	11.3 (1.0)*
Vitality	48.8 (16.8)	4.1 (5.2)	42.5 (20.0)	1.9 (4.2)	34.2 (17.0)	-0.3 (2.2)	38.5 (18.6)	5.6 (1.8)*	19.3 (15.7)	6.9 (1.1)	20.3 (17.3)	11.7 (0.9)***

*P < 0.05 vs. placebo; **P < 0.01 vs. placebo; ***P < 0.001 vs. placebo; ^a ^*P *≤ 0.1 therapy-by-fatigue subgroup interaction.

BL, baseline; SD, standard deviation; SE, standard error; BPI, brief pain inventory; FIQ, fibromyalgia impact questionnaire; SF-36, Medical Outcomes Study Short Form-36.

#### Fibromyalgia impact questionnaire

For the FIQ pain score, within the severe subgroup there was a significant difference (*P *< 0.001) in the mean change (standard error (SE)) from baseline to endpoint between the duloxetine (-2.1 (0.1)) and placebo (-1.4 (0.1)) groups (Table 2). Within the severe subgroup, there were also significant differences in the mean change from baseline to endpoint between the duloxetine and placebo groups for the FIQ total score, physical impairment score, interference with work, stiffness, anxious, and depression scores. For the FIQ tiredness score, there was no significant difference in the mean change (SE) from baseline to endpoint between the duloxetine and placebo groups, within any of the tiredness subgroups (mild 2.4 (0.7) (duloxetine), 1.6 (0.8) (placebo), moderate -0.2 (0.2) (duloxetine), 0.1 (0.3) (placebo), and severe -1.8 (0.1) (duloxetine), -1.6 (0.1) (placebo)). Other FIQ measures are also reported in Table 2.

The number of patients with 30% improvement in tiredness at endpoint was 257 (33.9%) in the duloxetine group and 150 (29.0%) in the placebo group (*P *= 0.067). The number of patients with 50% improvement in tiredness at endpoint was 173 (22.8%) in the duloxetine group and 93 (18.0%) in the placebo group (*P *= 0.042). No significant differences were observed between the duloxetine and placebo groups in the number of patients with 30% worsening in tiredness at endpoint (duloxetine, n = 62 (8.2%), placebo, n = 41 (7.9%); *P *= 0.917) or in the number of patients with 50% worsening in tiredness at endpoint (duloxetine, n = 46 (6.1%), placebo, n = 27 (5.2%); *P *= 0.542).

#### Patient global impressions of improvement

We found differences between the duloxetine-treated and placebo-treated patients in the frequencies of PGI-Improvement scores of 1 or 2 (patients feeling very much better or much better) across two of the three tiredness subgroups. There were significantly greater numbers of patients in the duloxetine, compared with the control group, who reported global positive improvements in both the severe tiredness subgroup (duloxetine, *n *= 233 (38.26%); placebo, *n *= 87 (21.07%); *P *< 0.001), and moderate tiredness subgroup (duloxetine, *n *= 45 (35.71%); placebo, *n *= 18 (21.95%); *P *= 0.044). This difference was not found in the mild tiredness subgroup (duloxetine, *n *= 15 (53.57%), placebo, *n *= 7 (36.84%); *P *= 0.373).

#### Medical outcomes study short form-36

Within the severe subgroup, there was a significant difference (*P *< 0.05) in the mean change of the SF-36 scores from baseline to endpoint between the duloxetine and placebo groups on the health outcome measures of the SF-36 mental component summary; bodily pain, general health, mental health, physical functioning, role-emotional, role-physical, social functioning, and vitality (Table 2).

### Treatment-by-tiredness subgroup interaction

The effect of duloxetine on the BPI average pain score was consistent across patients with mild, moderate, or severe tiredness (*P *> 0.1 for treatment-by-tiredness subgroup interaction). For the FIQ pain score, there was no significant therapy-by-tiredness subgroup interaction (*P *= 0.74). The effect of duloxetine on the FIQ total score, physical impairment score, interference with work score, pain score, tiredness score, rested score, stiffness score, and depression score was consistent across patients with mild, moderate, or severe tiredness (*P *> 0.1 for treatment-by-tiredness subgroup interaction). No significant therapy-by-tiredness subgroup interaction in the PGI-Improvement score was observed among the three tiredness subgroups (*P *= 0.908). The effects of duloxetine on the health outcome measures of the SF-36 mental component summary, physical component summary, bodily pain, general health, mental health, physical functioning, role-emotional, role-physical, social functioning, and vitality were consistent across patients with mild, moderate, or severe tiredness (*P *> 0.1 for treatment-by-tiredness subgroup interaction).

### Treatment-emergent adverse events

A significant (*P *< 0.1) treatment-by-tiredness interaction in the percentage of patients with one TEAE or more was observed. Significant treatment-by-tiredness interactions were found for the percentage of patients with the following TEAEs: hypoaesthesia, arthralgia, cough, myalgia, bronchitis, influenza, palpitations, restless leg syndrome, and osteoarthritis (Table 3). Nausea was the most common TEAE, with an occurrence of 28% (duloxetine) and 15% (placebo) in the mild subgroup, 29% (duloxetine) and 15% (placebo) in the moderate subgroup, and 30% (duloxetine) and 11% (placebo) in the severe subgroup. However, there was no significant treatment-by-tiredness subgroup interaction (*P *> 0.1) in the percentage of patients with nausea. Comprehensive pooled safety analyses for these four studies have been performed and previously reported [21].

**Table 3:** Treatment-emergent adverse events (%) with significant treatment-by-tiredness subgroup interaction

	*P*-value (treatment-by-tiredness subgroup)	Mild subgroup		Moderate subgroup		Severe subgroup	
		Placebo (n = 20) (%)	Duloxetine (n = 29) (%)	Placebo (n = 83) (%)	Duloxetine (n = 133) (%)	Placebo (n = 430) (%)	Duloxetine (n = 634) (%)
Patients with ≥1 TEAE	0.040	90.0	96.6	73.5	91.0	77.7	82.0
Hypoaesthesia	0.094	10.0	0.0	1.2	1.5	1.2	2.1
Arthralgia	0.008	10.0	0.0	9.6	2.3	2.8	3.8
Cough	0.034	15.0	0.0	1.2	3.0	2.6	3.2
Myalgia	0.081	10.0	3.5	0.0	3.8	1.6	3.3
Bronchitis	0.014	5.0	0.0	3.6	0.0	2.3	3.3
Influenza	0.019	0.0	3.5	6.0	0.8	1.4	2.5
Palpitations	0.085	0.0	3.5	4.8	1.5	2.5	1.2
Restless leg syndrome	0.030	5.0	0.0	0.0	3.0	1.4	0.6
Osteoarthritis	0.029	10.0	0.0	3.6	0.0	0.7	0.8

TEAE, treatment-emergent adverse event.

## Discussion

In a previously conducted pooled analysis of these same four randomized, double-blind, placebo-controlled trials in patients with fibromyalgia, almost half (47.7%) of the patients treated with duloxetine had a 30% or greater reduction in BPI average pain, and 35.3% of patients had a 50% or greater reduction in pain [22]. Among placebo-treated patients, 32.1% had a 30% or greater reduction in BPI average pain, and 22.2% had a 50% or greater reduction [22]. A previous pooled analysis of these four clinical trials [22] demonstrated that a significantly greater proportion of patients treated with duloxetine versus placebo reported feeling very much better or much better. More than one third (38.4%) of duloxetine-treated patients reported feeling much better and less than one quarter (21.7%) of the placebo-treated patients reported feeling much better, based on PGI-Improvement scores.

Several double-blind, placebo-controlled studies have shown that duloxetine, compared with placebo, more frequently produces clinically-significant improvements in pain and functional ability [8-11]. A pooled analysis of these studies showed that duloxetine was statistically superior to placebo with respect to improvement on FIQ total scores and all SF-36 domains [22]. Furthermore, the pooled data indicated that those patients who achieve 50% or greater improvement in tiredness were more likely to have received duloxetine than placebo [22].

In the pooled analysis of four placebo-controlled trials in patients with fibromyalgia reported here, the efficacy of duloxetine did not vary as a function of baseline ratings of tiredness, as defined by the FIQ tiredness rating.

At baseline, the distribution of tiredness severity in the duloxetine and placebo groups were less than 4% of patients in the mild subgroup and more than 90% of total patients in the moderate to severe subgroups. This is not surprising, as a previous study has reported that up to 90% of patients with fibromyalgia report moderate to severe fatigue levels [12].

The rates of clinically significant (≥30% and ≥50%) improvement in BPI average pain were similar across the three tiredness subgroups, demonstrating that the efficacy of duloxetine in the treatment of pain in patients with fibromyalgia is not associated with baseline tiredness levels.

Analyses of the PGI-Improvement produced findings similar to those for BPI average pain ratings. As with the other efficacy measures reported here, there was no significant therapy-by-tiredness subgroup interaction on the measure of PGI-Improvement. Baseline tiredness levels were not significantly associated with the effects of duloxetine on the patients' reports of functional ability using the FIQ total score and the FIQ measures of physical impairment, interference with work, pain, stiffness, depression and the SF-36 mental and physical component summary scores, bodily pain, general health, mental health, physical functioning, role-emotional, role-physical, social functioning, and vitality.

Some limitations of this work should be considered. The results are based on four acute treatment trials of 12 weeks and the results may not generalize to a longer duration of treatment. In addition, sleep and fatigue/tiredness may have a correlation. Future studies should measure sleep quality and its effect on fatigue/tiredness in the fibromyalgia population. The results of these studies may not generalize to individuals with fibromyalgia symptoms diagnosed with Diagnostic and Statistical Manual of Mental Disorders-IV axis-one psychiatric disorder, rheumatic disease, or treatment-resistant patients, because such patients were excluded from the studies. Most of the patients included in these studies were middle-aged Caucasian women, which may limit generalization of these results to men and other individuals with fibromyalgia. The FIQ tiredness item is used to substitute the formal measure of fatigue, because the formal fatigue measure was not used in all four of the fibromyalgia studies. The FIQ tiredness item may not capture all aspects of fatigue as a formal fatigue scale would. It is worth noting that question number 20 of the Beck depression inventory refers to fatigue and tiredness interchangeably. Lastly, the distribution of patients in the tiredness subgroups was skewed due to the large number of patients in the severe subgroup (up to 81%) compared with the moderate (up to 17%) and the mild (<4%) subgroups; therefore, the information on patients in the mild and moderate subgroups may not be as robust as that in the severe subgroup.

## Conclusions

This pooled analysis of four randomized placebo-controlled studies provides evidence that the efficacy of duloxetine on pain does not vary as a function of patients' baseline ratings of tiredness, thus implying that baseline tiredness may not need to be factored into models of efficacy regarding pain.

## Abbreviations

BPI: brief pain inventory; FIQ: fibromyalgia impact questionnaire; PGI-Improvement: patient global impressions of improvement; SE: standard error; SF-36: medical outcomes study short form-36; TEAE: treatment-emergent adverse events.

## Competing interests

Dr Bradley is a consultant for Eli Lilly, Pfizer, and Forest; has received grant/research support from the National Institutes of Health, the Agency for Healthcare Research and Quality, Eli Lilly, Pfizer, and the American Fibromyalgia Syndrome Association; has received honoraria from Eli Lilly, Pfizer, Forest, and the Society for Women's Health Research; is a member of the speakers/advisory board for Eli Lilly and Company; and has received royalties from UpToDate Rheumatology. Dr Bennett is on advisory boards for Eli Lilly and Company, Schwarz, Jazz and Pfizer. Dr. Russell has received honorarium as a consultant and speaker for Pfizer, Eli Lilly and Company, Jazz, Forest Laboratories, and Grunenthal, and as a consultant for Allergan. He has received honorarium for being a speaker on the medical advisory board for Pfizer, Eli Lilly and Company, Jazz, Pierre Fabre, Nicox, Daiichii Sankyo, and Ortho McNeil Jannssen. He has received funding as a principal investigator for Pfizer, as a local principal local investigator for Eli Lilly and Company, Allergan, and Schwarz/UCB, and as a lead investigator for Jazz, Grunenthal, and Autoimmune Technologies. Dr. Russell is the editor of the *Journal of Musculoskeletal Pain *and receives from the publisher a royalty for his work in that capacity. He is a member of the International MYOPAIN Society Board (non-remunerative position). Dr. Russell has never owned a pharmaceutical company, or owned stock in a pharmaceutical company that he is aware of, or worked as an employee for a pharmaceutical company. Dr Moldofsky is the principal investigator at a research site, the Toronto Psychiatric Research Foundation, a not for profit corporation that has received funds for participating in a research study, which is sponsored by Eli Lilly and Company. Dr. Moldofsky has received research support from Eli Lilly, Krele, Pfizer, Pierre Fabre, Sanofi Aventis, and Schering Plough; has acted as a consultant for Boehringer Ingleheim, Eli Lilly, Jazz Pharmaceuticals, Krele, Lundbeck, Merck, Paladin Labs, Pfizer, Pierre Fabre, Sanofi Aventis, Schering Plough, and Valeant; and was on the speaker's bureau for Pfizer. Drs Wohlreich, Chappell, Wang, and D'Souza are employees and stockholders of Eli Lilly and Company.

## Authors' contributions

Each of the authors listed have made substantive intellectual contributions to this study. LB, RB, IJR, MMW, ASC, FW, DND, and HM have made substantial contributions to analysis and interpretation of data, have been involved in drafting the manuscript and/or revising it critically for important intellectual content and have given final approval of the version to be published.

## References

[B1] Wolfe F Ross K Anderson J Russell IJ Hebert L The prevalence and characteristics of fibromyalgia in the general population Arthritis Rheum 1995 38 19 28 10.1002/art.1780380104 7818567

[B2] Bennett RM Jones J Turk DC Russell IJ Matallana L An internet survey of 2,596 people with fibromyalgia BMC Musculoskelet Disord 2007 8 27 10.1186/1471-2474-8-27 PMC1829161 17349056

[B3] Wolfe F Smythe HA Yunus MB Bennett RM Bombardier C Goldenberg DL Tugwell P Campbell SM Abeles M Clark P Fam AG Farber SJ Fiechtner JJ Franklin CM Gatter RA Hamaty D Lessard J Lichtbroun AS Masi AT McCain GA Reynolds WJ Romano TJ Russell IJ Sheon RP The American College of Rheumatology 1990 criteria for the classification of fibromyalgia. Report of the multicenter criteria committee Arthritis Rheum 1990 33 160 172 10.1002/art.1780330203 2306288

[B4] Seltzer Z Dubner R Shir Y A novel behavioral model of neuropathic pain disorders produced in rats by partial sciatic nerve injury Pain 1990 43 205 218 10.1016/0304-3959(90)91074-S 1982347

[B5] Kim SH Chung JM An experimental model for peripheral neuropathy produced by segmental spinal nerve ligation in the rat Pain 1992 50 355 363 10.1016/0304-3959(92)90041-9 1333581

[B6] Goldstein DJ Lu Y Detke MJ Hudson J Iyengar S Demitrack MA Effects of duloxetine on painful physical symptoms associated with depression Psychosomatics 2004 45 17 28 10.1176/appi.psy.45.1.17 14709757

[B7] Goldstein DJ Lu Y Detke MJ Lee TC Iyengar S Duloxetine vs. placebo in patients with painful diabetic neuropathy Pain 2005 116 109 118 10.1016/j.pain.2005.03.029 15927394

[B8] Arnold LM Lu Y Crofford LJ Wohlreich M Detke MJ Iyengar S Goldstein DJ A double-blind, multicenter trial comparing duloxetine with placebo in the treatment of fibromyalgia patients with or without major depressive disorder Arthritis Rheum 2004 50 2974 2984 10.1002/art.20485 15457467

[B9] Arnold LM Rosen A Pritchett YL D'Souza DN Goldstein DJ Iyengar S Wernicke JF A randomized, double-blind, placebo-controlled trial of duloxetine in the treatment of women with fibromyalgia with or without major depressive disorder Pain 2005 119 5 15 10.1016/j.pain.2005.06.031 16298061

[B10] Chappell AS Bradley LA Wiltse C Detke MJ D'Souza DN Spaeth M A six-month double-blind, placebo-controlled, randomized clinical trial of duloxetine for the treatment of fibromyalgia Int J Gen Med 2009 1 91 102 10.2147/ijgm.s3979 PMC2840539 20428412

[B11] Russell IJ Mease PJ Smith TR Kajdasz DK Wohlreich MM Detke MJ Walker DJ Chappell AS Arnold LM Efficacy and safety of duloxetine for treatment of fibromyalgia in patients with or without major depressive disorder: Results from a 6-month, randomized, double-blind, placebo-controlled, fixed-dose trial Pain 2008 136 432 444 10.1016/j.pain.2008.02.024 18395345

[B12] Goldenberg DL Simms RW Geiger A Komaroff AL High frequency of fibromyalgia in patients with chronic fatigue seen in a primary care practice Arthritis Rheum 1990 33 381 387 10.1002/art.1780330311 2317224

[B13] Wolfe F Hawley DJ Wilson K The prevalence and meaning of fatigue in rheumatic disease J Rheumatol 1996 23 1407 1417 8856621

[B14] Fishbain DA Cole B Cutler RB Lewis J Rosomoff HL Fosomoff RS Is pain fatiguing? A structured evidence-based review Pain Med 2003 4 51 62 10.1046/j.1526-4637.2003.03008.x 12873278

[B15] Feuerstein M Carter RL Papciak AS A prospective analysis of stress and fatigue in recurrent low back pain Pain 1987 31 333 344 10.1016/0304-3959(87)90162-X 2962055

[B16] Stone P Richards M A'Hern R Hardy J A study to investigate the prevalence, severity and correlates of fatigue among patients with cancer in comparison with a control group of volunteers without cancer Ann Oncol 2000 11 561 567 10.1023/A:1008331230608 10907949

[B17] Burckhardt CS Clark SR Bennett RM The fibromyalgia impact questionnaire: development and validation J Rheumatol 1991 18 728 733 1865419

[B18] Cleeland CS Ryan KM Pain assessment: global use of the Brief Pain Inventory Ann Acad Med Singapore 1994 23 129 138 8080219

[B19] Krupp LB LaRocca NG Muir-Nash J Steinberg AD The fatigue severity scale. Application to patients with multiple sclerosis and systemic lupus erythematosus Arch Neurol 1989 46 1121 1123 2803071 10.1001/archneur.1989.00520460115022

[B20] Mendoza TR Wang XS Cleeland CS Morrissey M Johnson BA Wendt JK Huber SL The rapid assessment of fatigue severity in cancer patients: use of the Brief Fatigue Inventory Cancer 1999 85 1186 1196 10.1002/(SICI)1097-0142(19990301)85:5<1186::AID-CNCR24>3.0.CO;2-N 10091805

[B21] Choy EH Mease PJ Kajdasz DK Wohlreich MM Crits-Christoph P Walker DJ Chappell AS Safety and tolerability of duloxetine in the treatment of patients with fibromyalgia: pooled analysis of data from five clinical trials Clin Rheumatol 2009 28 1035 1044 10.1007/s10067-009-1203-2 19533210 PMC2721139

[B22] Arnold LM Clauw D Wohlreich MM Wang F Ahl J Gaynor PJ Chappell AS Efficacy of duloxetine in patients with fibromyalgia: pooled analysis of four placebo-controlled clinical trials Prim Care Companion J Clin Psychiatry 2009 11 237 244 10.4088/PCC.08m00680 19956462 PMC2781036

